# Exploiting time series of Sentinel-1 and Sentinel-2 to detect grassland mowing events using deep learning with reject region

**DOI:** 10.1038/s41598-022-04932-6

**Published:** 2022-01-19

**Authors:** Viacheslav Komisarenko, Kaupo Voormansik, Radwa Elshawi, Sherif Sakr

**Affiliations:** 1 Institute of Computer Science, University of Tartu, Tartu, Estonia; 2 Tartu Observatory, University of Tartu, Tartu, Estonia; 3 KappaZeta Ltd., Tartu, Estonia

**Keywords:** Ecology, Environmental sciences

## Abstract

Governments pay agencies to control the activities of farmers who receive governmental support. Field visits are costly and highly time-consuming; hence remote sensing is widely used for monitoring farmers’ activities. Nowadays, a vast amount of available data from the Sentinel mission significantly boosted research in agriculture. Estonia is among the first countries to take advantage of this data source to automate mowing and ploughing events detection across the country. Although techniques that rely on optical data for monitoring agriculture events are favourable, the availability of such data during the growing season is limited. Thus, alternative data sources have to be evaluated. In this paper, we developed a deep learning model with an integrated reject option for detecting grassland mowing events using time series of Sentinel-1 and Sentinel-2 optical images acquired from 2000 fields in Estonia in 2018 during the vegetative season. The rejection mechanism is based on a threshold over the prediction confidence of the proposed model. The proposed model significantly outperforms the state-of-the-art technique and achieves event accuracy of 73.3% and end of season accuracy of 94.8%.

## Introduction

Grasslands constitute a significantly large part of Europe’s agricultural area^1^. Grassland is an essential component in regulating the global carbon cycle^2^. The European Union has established the Common Agriculture Policy (CAP) that financially supports the maintenance of the grasslands^3^. CAP uses a measure called *Greening* that makes the direct payments more environmental-friendly. For landowners to receive financial support, they must maintain grasslands in good agricultural and environmental conditions. Monitoring mowing events is done by National Paying Agencies (NPA), responsible for verifying subsidy claims. Generally, monitoring is done by checking a limited number of grasslands through on-site field inspection, in addition to the visual interpretation of images obtained from a high-resolution satellite. Therefore, monitoring mowing events is very time-consuming and costly. Hence, finding a cost-effective method to provide mowing information over large spatial scales is essential. Remote sensing can be used as a cost and time-efficient solution for providing accurate information on mowing grasslands^4,5^, especially after the launch of the Copernicus program^6^. Using Earth Observation (EO) data enables NPAs to monitor much more grasslands, promotes the effective use of resources, and significantly reduces the number of fraudulent payments.

Research in grasslands and agriculture vegetation is generally categorised into the following categories: (a) identifying grasslands from other land types, (b) classification of grassland types, (c) monitoring grassland events. Remote sensing plays an essential role in many complex environments such as agriculture. Remote sensing is a well-established research topic^7,8^ and has a rich literature on grasslands^2,9^. Optical remote sensing offers a wide range of techniques for monitoring farming events^10,11^ and management practices^12^. Typical approaches for assessing the state of grasslands and their dynamics rely on the use of optical satellite data combined along with field spectral measurements and *in situ* data^13,14^. Methods that rely on time series are favourable due to temporal differences caused by plant phenology and management practices. However, the availability of optical data during the growing season is limited due to cloud cover that may result in shorter or longer gaps in time series of images which motivates studying other remote sensing techniques for monitoring grasslands^15^. Laser scanning produces reliable vegetation height estimates^16^ however, it is too expensive for frequent monitoring of large areas. Spaceborne synthetic aperture radar (SAR) data is considered an effective solution for monitoring agriculture activities^17^, as the SAR signal can penetrate clouds in various weather conditions while providing continuous data covering a large area. Due to the availability of C-band SAR with a wavelength of $\thickapprox 5$ cm and X-band SAR with a wavelength of $\thickapprox 3$ cm, they have been used for monitoring grasslands and agricultural crops^18,19^. In addition, C-band SAR is sensitive to surface roughness^20,21^ and the dominant scattering mechanism over grassland in C-band is volume scattering^22,23^. Hence, it is shown that there is a linear relation between grassland vegetation height and the C-band backscatter^24^. Tamm et al.^25^ used Sentinel-1 interferometric coherence for studying mowing events on agricultural grassland and showed that the median coherence values for VV and VH polarisation after mowing events were statistically significantly higher than that before mowing. SAR data might not completely substitute optical data, but it is a valuable supplement. Schuster et al.^26^ presented a technique based on backscatter analysis of the TerraSAR-X time series for detecting the mowing events for natural habitats. Voormansik et al.^27^ studied the problem of detecting mowing events using multi-temporal, dual polarimetric x-band SAR. Although there is a rich literature on monitoring agricultural activities, there are very few studies considering both optical and SAR time-series data^28,29^. Although most studies on data combination demonstrate significant improvements in monitoring agriculture events, there is a lack of proper methods in detecting mowing events.

Although machine learning achieved notable performance in different applications, only a limited number of studies used machine learning in detecting mowing events^30–32^. Barret et al.^19^ presented a model for classifying different types of grassland using multi-temporal SAR data. The model achieved an accuracy of 88.7% and 97.9% using single and multiple frequencies, respectively. Several studies used multi-frequency SAR data in identifying grasslands from other land cover types^33–35^. Other studies focused on combining single polarisation single frequency SAR data and optical imagery in identifying grasslands^36,37^. Hong et al.^17^ used only SAR data for identifying grasslands from other land types and achieved a classification accuracy of 64%. On the other hand, Smith and Buckley^38,39^ classify different types of grasslands and crops using Freeman–Durden decomposition on a multi-temporal fully polarimetric RADARSAT-2 data set and achieved a classification accuracy of 78%.

In classification with a reject region option, a classifier can abstain from the prediction in case of uncertainty which is essential in many applications, especially critical applications such as autonomous driving and medical diagnosis. The idea of the reject region was introduced a long time ago by Chow^40^. He created an optimal rejection rule that optimises classification error for a binary classification problem. More specifically, instances are rejected when the distance to a discrimination plan is lower than a particular set threshold. There is a rich literature on the topic of the reject option that mainly focuses on rejection mechanisms for different hypothesis classes and machine learning algorithms^41–44^. The concept of the reject option has been rarely considered for neural networks and deep neural networks^45,46^.

Since 2015, Tartu Observatory^47^ and its spin-off Kappazeta Ltd^48^ have actively pursued the goal of creating a system capable of detecting grassland management practices based on Sentinel-1 and Sentinel-2 data. Kappazeta develops solutions for automated monitoring of mowing events on grassland. Such solutions have been operational in Estonia since 2017. In addition, there are successful trials in Sweden, Denmark and Poland to enhance the existing cutting and grazing detection techniques taking into account the varying climatic, ecological and agricultural conditions. In this work, we introduce a generic machine learning workflow to detect mowing events. We developed a deep learning model with an integrated reject option for detecting mowing events using time-series of Sentinel-1 SAR and Sentinel-2 optical images covering 2000 fields in Estonia during vegetative season 2018. The combination of Sentinel-1 and Sentinel-2 images reduce the problems resulting from frequent cloud cover and lack of sunlight and yield additional information^49^. The proposed machine learning pipeline is generic and can be used in other countries such as Denmark and Sweden. In addition, the proposed pipeline can also be transferred to the different earth monitoring applications. Existing studies for detecting mowing events mainly consider the problem of predicting whether a field is mown or not mown and do not take advantage of the sequential nature of data. In this work, we focus on detecting mowing events from time series obtained from Sentinel-2 optical images and Sentinel-1 6-day interferometric coherence time series, which is conceptually much more complex task and has many critical applications (e.g. real-time system for sending notifications to farmers to perform mowing events when the system detects that their fields are not mown in time). In addition, the proposed approach is based on 1-D CNN architecture to handle sequential data, which means the temporal proximity of the data will be fully exploited. The paper is organised as follows. “Materials and methods” section outlines the dataset used in this study in addition to the pre-processing methods and algorithm used to detect mowing events. “Results” and “Discussion” sections contain results and discussion. Conclusion and potential future work is presented in “Conclusion” section.

## Materials and methods

### Study area and dataset

**Figure 1:**
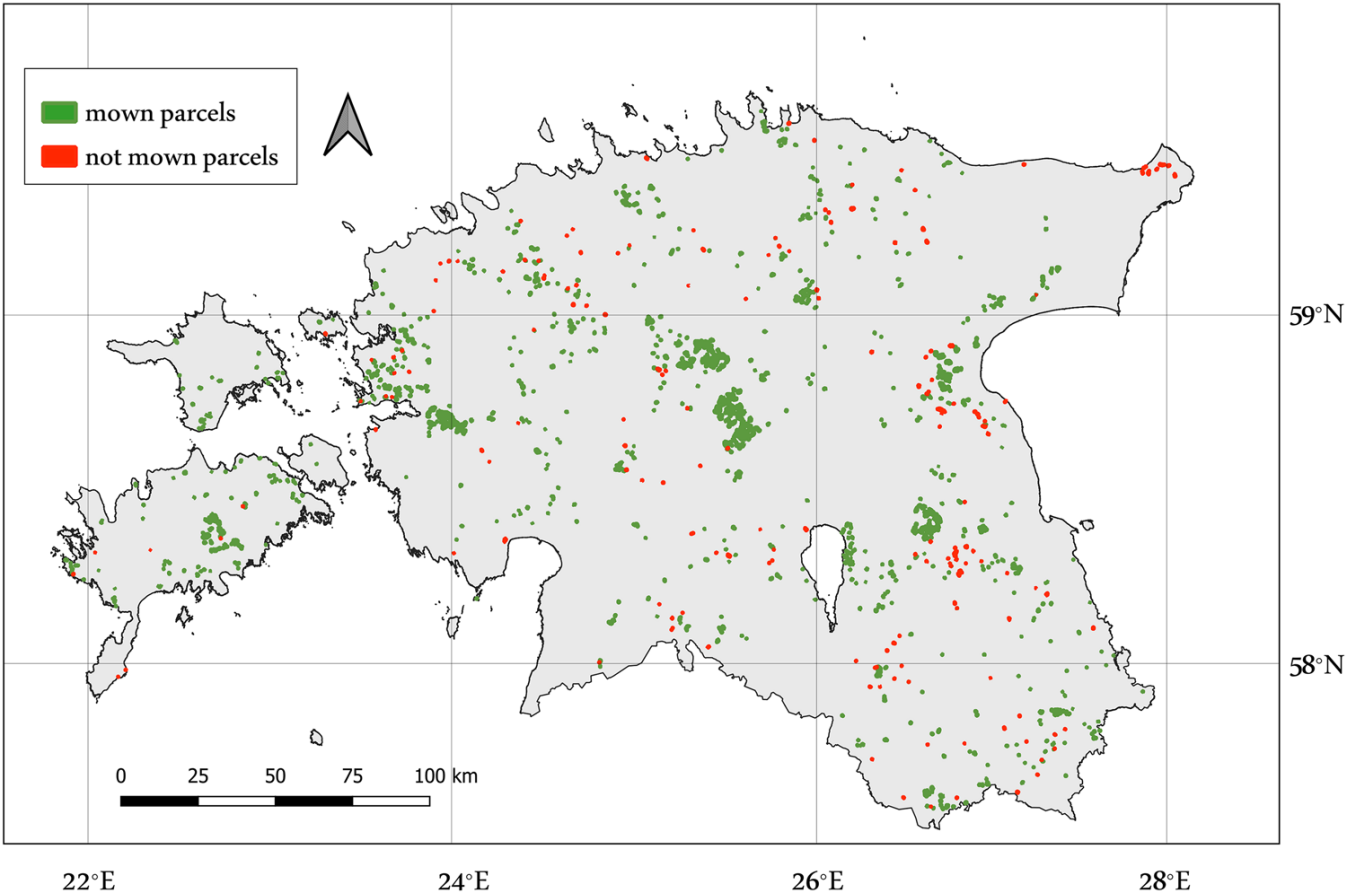
Geographic distribution of events used in this study (This map was created by QGIS version 3.16, which can be accessed on https://qgis.org/en/site/).

The study area covers all Estonia located between 57.5$^\circ$ N, 21.5$^\circ$ E and 59.8$^\circ$ N, 28.2$^\circ$ E. The study area is relatively flat with no steep slopes and altitudes ranging between 0 and 200m above the sea level. Data about events were collected directly from field books that contained information about the mowing activity’s start and end date and the covered area. Considering the main agricultural areas of the country, we consider 2000 fields in which events are geographically evenly distributed across all Estonia, as shown in Fig. 1. In total, data about 1800 mowing and 200 non-mown events were collected in 2018, based on manual labelling. During manual labelling, the specific mowing days were labelled based on the following: a) information recorded by farmers in field books regarding mowing days, b) domain experts knowledge about the most probable days for mowing based on the climate, weather, and field conditions, c) rapid decrease in the Normalized Difference Vegetation Index (NDVI) and rapid increase in the coherence compared to past measurements. The average field size is 6.0ha, and around 95% of the fields were mown during the year. 90% of the fields are in the range of $0.5-10$ha. The greatest density of the fields is located in Lääne-Viru, Tartu and Jõgeva countries. Grassland parcels vector layer is provided by Estonian Agricultural Registers and Information Board (ARIB)^50^. The satellite imagery used in the study is from Copernicus program that provides free open Earth observation data to help service providers, public authorities, and international organizations improve European citizens’ quality of life.

### Sentinel-1 and Sentinel-2 data

For Sentinel-1 data, in total, 400 S1A/BSLCIW products acquired between 1st of May 20017 and 30th of October 2018, were processed. 87 products were from relative orbit number (RON)160, 62 from RON131, 84 from RON87, 93 from RON58, and 60 from RON29. These were organised into S1A/S1B 6-day pairs. Sentinel-2 provides high spatial resolution optical imagery to perform terrestrial observations with global coverage of the Earth’s land surface. Sentinel-2 data is provided by the European Space Agency (ESA) together with a cloud mask, which can filter clouds on the image with moderately good accuracy. 400 Sentinel-2A and -2B L2A products acquired between 1 May 2017 and 30 October 2018 were processed. Each Sentinel-2 image is a maximum of three days off from the closest Sentinel-1 image. Only the NDVI was derived from Sentinel-2. NDVI has been widely used in the classification of grassland^24,51^ and that is mainly due to its ability in limiting spectral noise. The spatial resolution of the derived Sentinel-2 NDVI feature is 10 m.

### Methods

**Figure 2:**
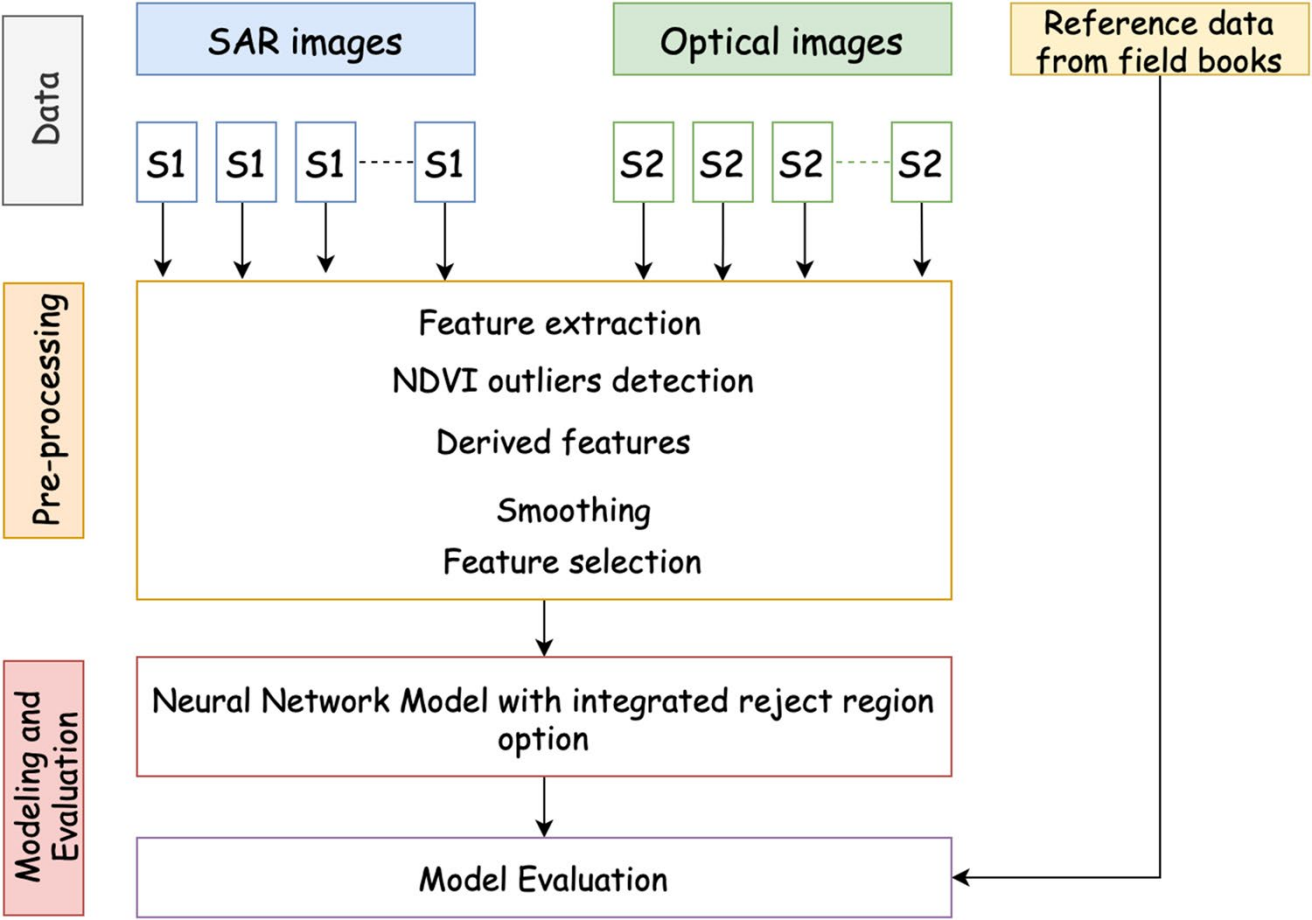
Flowchart of the proposed approach to detect mowing events.

The goal of the analysis is to detect mowing events from Sentinel-1 (S-1) and Sentinel-2 (S-2) data. For this, coherence time series were calculated about every field in the database about the event. Average coherence of a field, imaging geometry parameters, imaging time and average NDVI were stored in a database. The database formation process involved preprocessing many satellite images where average coherence and NDVI value was calculated for every parcel for every available date (constrained by image availability and cloud cover). The overall scheme of the proposed methodology is illustrated in Fig. 2. First, the time-series data from S-1 and S-2 images are preprocessed. Then, the most important features are used in a deep neural network to predict mowing events. The model has a reject region option that enables the model to abstain from the prediction in case of uncertainty, which increases trust in the model.

We used the Sentinel Application Platform (SNAP) toolbox for processing S-1 data. More specifically, we followed the same following pre-processing steps in^16^: apply orbit file, back-geocoding (using Shuttle Radar Topography Mission (SRTM) data), coherence calculation, deburst, terrain correction, and reprojection to the local projection (EPSG:3301). Lastly, we resampled the data to 4m resolution to preserve the maximum spatial resolution and square-shaped pixels. Because the study areas’ terrain is relatively flat, there are few topographic distortions in the SAR data. Each swath’s coherence was calculated independently. Only pixels totally inside the parcel boundaries (including the average window used for coherence computation) were utilized to calculate results, and any interference beyond the parcel limits was discarded. Pair-wise coherence was calculated with 6-day time step. The data was stored into a database using a forward-looking convention: coherence regarding date X refers to the coherence between S-1 images over the period between date X and X + 6 days. For preprocessing S-2 data, L1C and L2A Sentinel-2 products were obtained through Copernicus Open Access Hub^6^. Next, a rule-based cloud mask solution was applied^52^. Finally, the fourth and eighth bands were extracted to compute NDVI values.

#### Feature extraction from Sentinel-1 data

Coherence is a normalized measure of similarity between two consecutive (same relative orbit) S-1 images. Interferometric 6 day repeat pass coherence in VV polarization (cohvv), and coherence in VH polarization (cohvh) are chosen features as they are shown to be sensitive to changes in vegetation and agricultural events^25^. The shorter the time interval after the mowing event and the first interferometric acquisition, the higher the coherence value. Generally, up to 24 to 36 days after a mowing event, coherence stays relatively high. Precipitation caused the coherence to drop, which disturbs the detection of a mowing event. The spatial resolution of the S-1 6-day repeat pass interferometric coherence is 70 m. Given two S-1 images $s_{1}$ and $s_{2}$, coherence is calculated as follows:

1 $$\begin{aligned} \wp =\frac{|\langle s_{1}s*_{2}\rangle |}{\sqrt{\langle s_{1}s*_{1}\rangle | \langle s_{2}s*_{2}\rangle |}} \end{aligned}$$

where $|\langle s_{1}s*_{2}\rangle |$ is the absolute value of the spatial average of the complex conjugate product.

Coherence between two S-1 images $s_1$ and $s_2$ reaches its maximum value of 1 when both images have the same position and physical characteristics of the scatters. In contrast, the coherence value declines when the position or properties of the scatters change.

#### Feature extraction from Sentinel-2 data

NDVI is related to the amount of live green vegetation. Generally, NDVI increases and decreases over the season, indicating the natural growth decay of vegetation, while the significant drops in the NDVI indicate an agricultural event such as mowing. NDVI is derived from S2 images and is calculated as follows:

2 $$\begin{aligned} NDVI=\frac{band8 - band4}{band8 + band4} \end{aligned}$$

**Figure 3:**
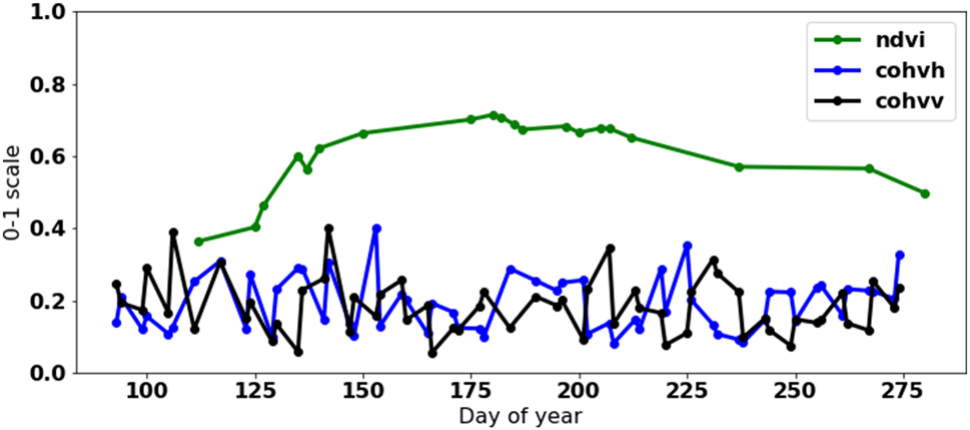
Typical signature of *NDVI* and coherence in VV and VH polarisation for non mown field during the year.

**Figure 4:**
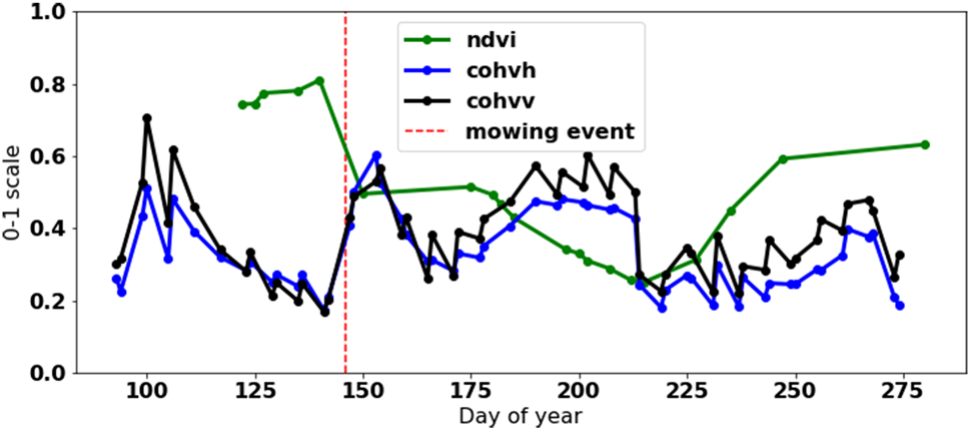
Field with single mowing event during the year.

**Figure 5:**
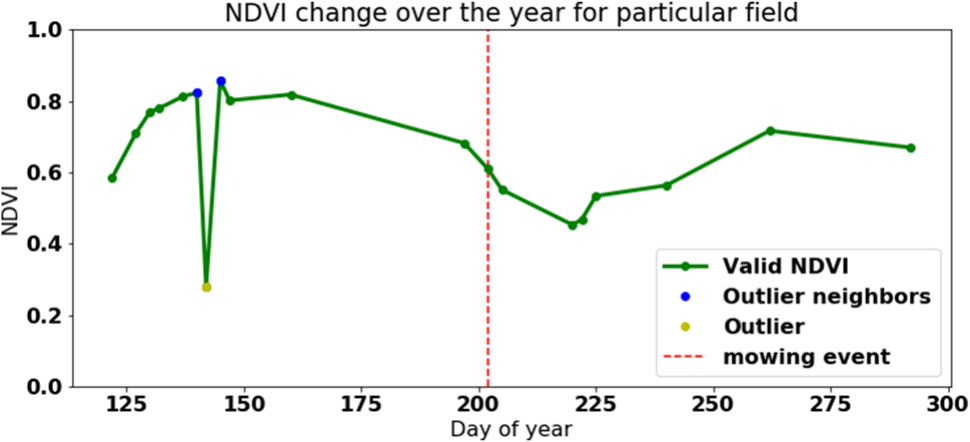
NDVI measurement for a field example with a single mowing event during the season.

Figures 3, 4 and 5 show different samples of mown and non mown fields. NDVI measurements are green, cohvh and cohvv are blue and black, respectively. For non mown field, the typical signature of NDVI during the year is shown in Fig. 3. For non mown field, the typical signature of NDVI during the season is a half-oval curve; coherence is not stable but remains at almost the same level without apparent trend changes, as shown in Fig. 3. An example of a field with a single mowing event during the season is shown in Fig. 4. A mowing event is characterized by a rapid increase in both cohvh and cohvv and a sharp decrease in NDVI, as observed at day 150 (See Fig. 4). Forty days later, a similar signature is probably not due to a mowing event but likely caused by drought during summer.

Notably, NDVI measurements are irregular and relatively sparse. Around 75% of total NDVI measurements are invalid in Estonia, and the percentage is slightly lower in Southern Sweden and Denmark due to cloud cover. The Cloud mask indicates the percentage of cloud coverage and allows the cloudy and cloud-free pixels to be identified. Using the standard cloud mask technique by the European Space Agency (ESA) leads to outliers noticed in the sudden decrease in the NDVI. Figure 5 shows an extreme value of NDVI that is supposed to be an outlier due to high differences to the precedent and subsequent values. The outlier is marked with a yellow dot (NDVI=0.38), nearest previous (NDVI=0.75), and next (NDVI=0.78) measurements are marked with a blue colour.

#### Sentinel-1 and Sentinel-2 data preprocessing

**Figure 6:**
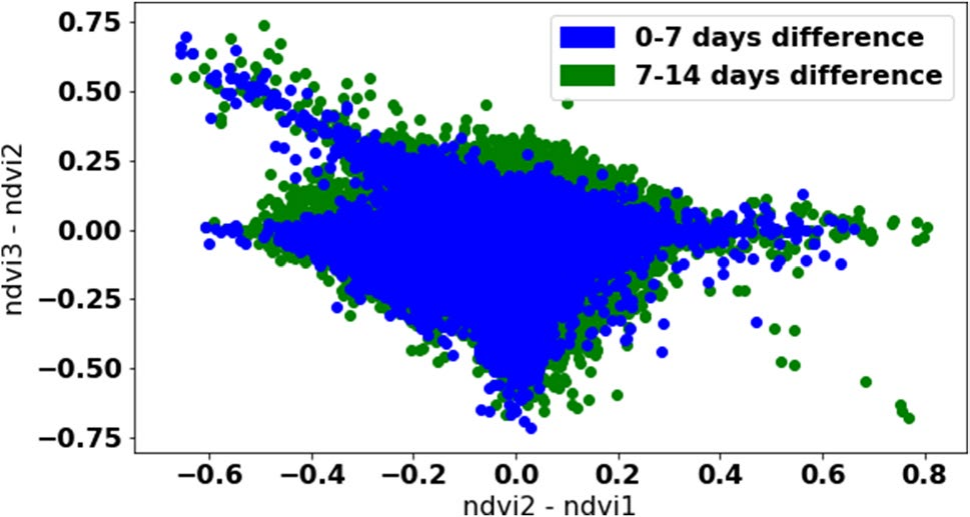
Scatter plot of NDVI triplets.

To detect NDVI outliers effectively, a good understanding of the data is needed. NDVI outliers due to cloud mask errors rarely co-occur together, and hence, they can be treated as independent events^53^. NDVI outliers are usually identified with a sudden drop to almost zero and do not form a sequence. It is enough to look at neighbouring measurements (one before and one after) to detect individual outliers. If the difference between the adjacent measurements is high, this is an outlier signature. Hence, outliers can be handled by iterating through every three consecutive NDVI measurements for a given field and checking the difference between the first and second values and between third and second values. Figure 6 shows the scatter plot of all three consecutive NDVI measurements. The Y-axis shows the difference between third and second NDVI values in a triplet, while X-axis represents the difference between second and first NDVI values in a triplet. Triplets with up to 7 days difference are shown in blue, and triplets from 7 to 14 days are shown in green. The points structure forms a rhombus shape with a small cloud of possible outliers in the upper left corner. To filter outliers from the list of actual mowing events, we only consider triplets within up to 10 days interval (as the mowing event signature can recover in 10 days). Knowing rhombus equation (the centre is approximately in (0, 0), and the side length is around 0.6), the filtering rule can be easily applied as follows:

3 $$\begin{aligned} ndvi\_3 - 2 \cdot ndvi\_2 + ndvi\_1 \ge 0.6 \end{aligned}$$

where ndvi_1, ndvi_2, and ndvi_3 are consecutive NDVI measurements within 10 days interval.

All outliers are removed, which represent around 0.1% of NDVI measurements.

Smoothing is an essential pre-processing step for noisy features. In this work, *cohvh* and *cohvv* features are smoothed using different techniques, including exponential moving average (EMA), moving average^54^, and Kalman filter^55^. Smoothing using moving average is done by taking the averages of raw data sequences. The length of the sequence over which we take the average is called the filter width. Table 1 shows the performance of moving average smoothing technique using different values for the filter width. The results show that the best AUC-ROC of 0.9671 is achieved at a filter size of 7. The Kalman filter produces estimates of the current state variables and their uncertainties. Once the outcome of the subsequent measurement is observed, these estimates are updated using a weighted average, giving more weight to estimates with higher certainty. The AUC-ROC achieved using Kalman filter is 0.962. The EMA is done by taking averages of sequences of data, in addition to assigning weights to every data point. More specifically, as values get older, they are given exponentially decreasing weights. The smoothed *cohvh* and *cohvv* EMA for *cohvh* and *cohvv* are calculated using a recursive definition (i.e., from its previous value) as follows:

4 $$\begin{aligned}&cohvh\_sm(cohvh_n, \alpha ) = \alpha \cdot (cohvh_n) + (1 - \alpha ) \cdot cohvh\_sm(cohvh_{n-1}, \alpha ) \end{aligned}$$

5 $$\begin{aligned}&cohvv\_sm(cohvv_n, \alpha ) = \alpha \cdot (cohvv_n) + (1 - \alpha ) \cdot cohvv\_sm(cohvv_{n-1}, \alpha ) \end{aligned}$$

where $cohvh\_sm(cohvh_{n-1}, \alpha )$: exponential moving average for end of $cohvh_{n-1}$. $cohvv\_sm(cohvv_{n-1}, \alpha )$: exponential moving average for end of $cohvv_{n-1}$. $\alpha$: a smoothing parameter.

The higher the smoothing parameter, the more it reacts to fluctuations in the original signal. The lower the smoothing parameter, the more the signal is smoothed. Experimentally, we found that the best value for $\alpha$ to achieve the best AUC-ROC of 0.968 is $\frac{1}{3}$ as shown in Table 2. The different smoothing techniques achieve comparable performance. EMA technique was selected as it achieves slightly higher performance.

**Table 1:** Performance of moving average smoothing using different filter width.

Filter width	AUC-ROC
3	0.953
5	0.964
7	0.9671
10	0.967

**Table 2:** Performance of EMA smoothing using different values of $\alpha$.

$\alpha$	AUC-ROC
$\frac{1}{6}$	0.965
$\frac{1}{5}$	0.965
$\frac{1}{4}$	0.966
$\frac{1}{3}$	**0.968**
$\frac{1}{2}$	0.967
$\frac{2}{3}$	0.967

Significant values are in [bold].

#### Derived features

New derived features from S-1 and S-2 are extracted to improve the performance of the machine learning model. The features were derived based on the following knowledge about mowing events: coherence tends to increase. In contrast, ndvi tends to decrease after mowing events and, many farmers perform mowing during the same time of the year due to the good weather conditions. Such knowledge was elaborated with the derived features. In the following, we will go through the list of derived features considered in this study. Mixed coherence is derived from S-1 features to capture the overall coherence trend. Mixed coherence is a non-linear combination of *cohvh* and *cohvv* and is calculated as follows:

6 $$\begin{aligned} Mixed\_coh = \sqrt{cohvh \cdot cohvv} \end{aligned}$$

The date is an important feature for the model to adapt, as it is more likely to have mowing events in the summer rather than in early spring, especially in Estonia. The normalized day of the year is calculated as normalization improves the training process of the neural network. Some methods normalize features during the training process, such as Batch Normalization used in this study^56^. However, neighbouring batches could have entirely different normalization variables (batch mean and variance). At the same time, DOY is a feature susceptible to small changes, e.g., mowing prediction on day 108 or 109 could have drastically different meaning (weekend or working day, day with sunny weather or day with heavy rain). It implies that unified normalization of the DOY feature before training could help avoid the unwanted impact of Batch normalization and possible gradient computation issues. The normalized day of the year is calculated as follows:

7 $$\begin{aligned} t = \frac{day\_of\_year}{365} \end{aligned}$$

where $day\_of\_year$ is the year’s day, which is a number between 1 and 365, January 1st is day 1.

In addition, we use another time feature *dt* to capture the gaps in time series. *dt* is defined to be the normalized difference in days between the current measurement and the previous one. Normalization was performed with min-max scaling. *dt* is calculated as follows:

8 $$\begin{aligned} dt = \frac{diff - min\_diff}{max\_diff - min\_diff} \end{aligned}$$

where $min\_diff$: the minimum difference in days between two previous consecutive measurements obtained from training data. $max\_diff$: the maximum difference in days between two previous consecutive measurements obtained from training data.

Since mowing is characterized by an increase in the coherence and decline in the NDVI, it is important to capture the difference in the values of features and/or slopes of the features’ curves. In the following, we summarize the list of original and derived features extracted from Sentinel-1 and Sentinel-2 included in this study.

- *ndvi* Normalized difference vegetation index, obtained from Sentinel-2.
- *cohvv* Coherence in VV polarization, Sentinel-1 feature.
- *cohvh* Coherence in VH polarization, Sentinel-1 feature.
- *t* Normalized day of the year when the measurement is obtained.
- *dt* Normalized difference in days between current and previous measurement. The data was interpolated with a daily grid, this feature differentiated between interpolated data and real data by capturing the difference between valid (not interpolated) measurements.
- *cohvv_sm* Smoothed *cohvv* with exponential mowing average (with parameter $\frac{1}{3}$).
- *cohvh_sm* Smoothed *cohvh* with exponential moving average (with parameter $\frac{1}{3}$).
- *mixed_coh* Harmonic mean of *cohvv* and *cohvh*. The harmonic mean is chosen as one of the simplest options of non-linear combination.
- *ndvi_diff* Difference between current and previous *NDVI* measurements. This feature captures the decrease in the ndvi, which is highly related to mowing detection.
- *cohvv_sm_diff* difference between current and previous $cohvv\_sm$ measurements. This feature captures the increase in the $cohvv\_sm$, which is highly related to mowing detection.
- *cohvh_sm_diff* difference between current and previous $cohvh\_sm$ measurements. This feature captures the increase in the $cohvh\_sm$, which is highly related to mowing detection.
- *ndvi_der * The slope of the line between previous and current *NDVI* values.
- *cohvh_sm_der* The slope of the line between previous and current $cohvh\_sm$ values. This feature captures the change in the smoothed *cohvh*.
- *cohvv_sm_der* The slope of the line between previous and current $cohvv\_sm$ values. This feature captures the change in the smoothed *cohvv*.

#### Feature selection

**Figure 7:**
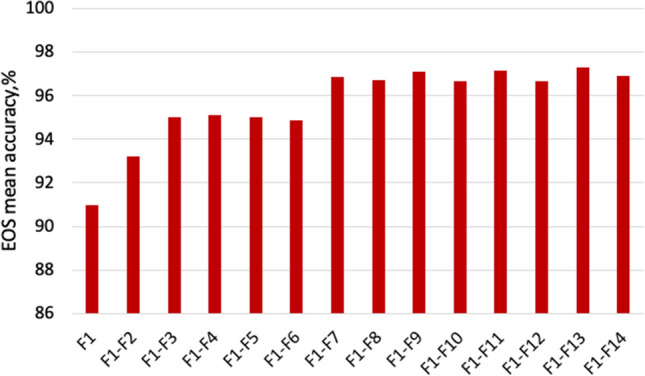
End of season accuracy for different number of features.

**Figure 8:**
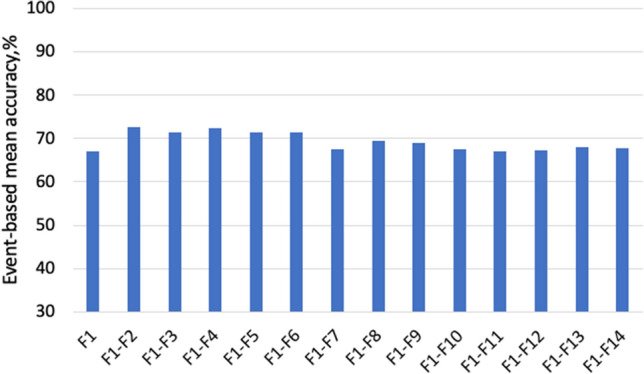
Event-based accuracy for different number of features.

The permutation feature importance measurement was introduced by Breiman^57^. The importance of a particular feature is measured by the increase in the model’s prediction error after we permuted the values of this feature, which breaks the relationship between the feature and the outcome. A feature is important if shuffling its values increases the model error and is less important otherwise. The importance of features considered in this study is ranked in Table 3. It is notable from Table 3 that the ordinal features are significantly more important than the derived ones. We used backwards elimination to select the optimal subset of features to be used by the machine learning model. More specifically, we start with all the features and then remove the least significant feature at each iteration, which improves the model’s overall performance. We repeat this until no improvement is observed on the removal of features. Figures 7 and 8 show that the end of season accuracy(EOS) and event accuracy, respectively, for training using a different subsets of the most important features. We refer to $F_{x}-F_{y}$ to be the set of important features from feature *x* to feature *y* in Table 3. Figure 7 shows that using only *ndvi* and $mixed_{coh}$ achieves EOS of 93%. Increasing the number of the most important features to 3 achieves a comparable performance to the best one, as shown in Fig. 7. The results show that using the *ndvi* and $mixed_{coh}$ achieve around 73% event accuracy while increasing the number of features, the performance declines as shown in Fig. 8. As an outcome of the feature selection process, the developed machine learning model used all the 14 features, shown in Table 3, that achieve the highest combined performance.

**Table 3:** Ranking features based on their performance.

Num	Feature name	Importance
1	ndvi	0.12
2	mixed_coh	0.08
3	cohvv	0.07
4	t	0.05
5	cohvv_sm	0.04
6	cohvh	0.04
7	cohvh_sm	0.04
8	ndvi_diff	0.02
9	cohvv_sm_diff	0.02
10	cohvh_sm_diff	0.02
11	dt	0.01
12	ndvi_derivative	− 3e−10
13	cohvh_sm_der	− 3e−10
14	cohvv_sm_der	− 3e−10

#### Machine learning model

**Figure 9:**
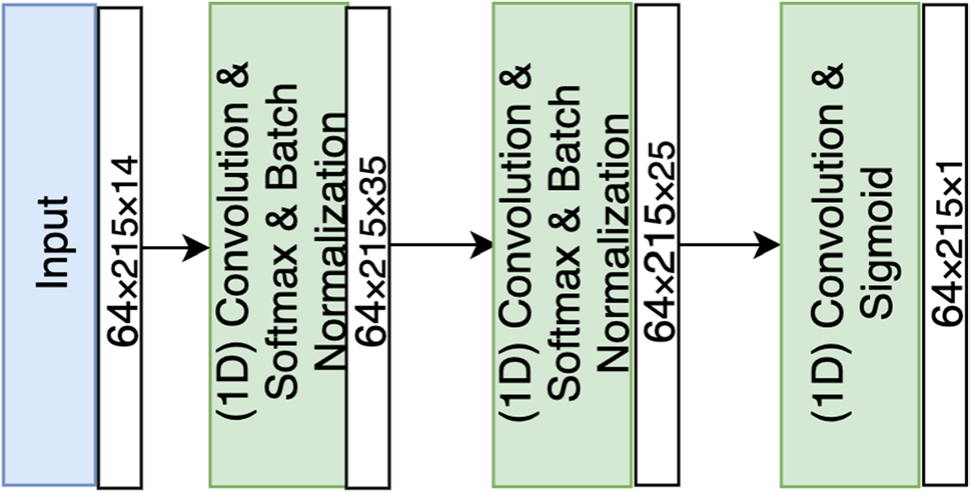
Architecture of the proposed model.

**Figure 10:**
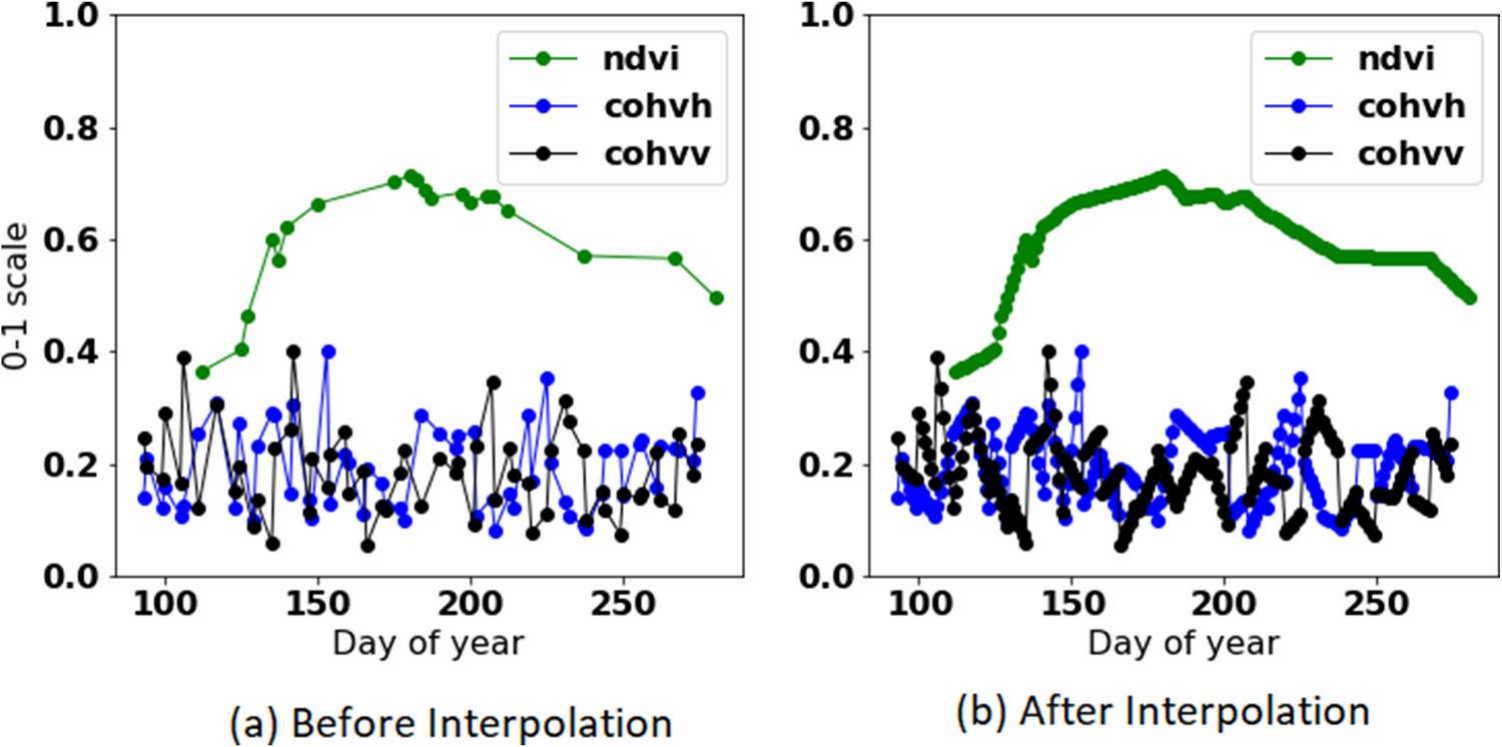
Time series mowing events before and after linear interpolation.

Each record in our dataset represents specific features about a field during one season at a particular time, in addition to the target variable (mown or non mown). In this work, we use a neural network to predict mowing events. We are interested only in observations during the vegetative season, so winter measurements are not included. More specifically, we only include the data in the vegetative season, which is almost the same across all Estonia from April till October (215 days). The dataset is partitioned into 64% for training, 20% for testing and 16% for validation. All training and testing were performed using TensorFlow^58^ deep learning framework with default parameters. The architecture of the neural network used is shown in Fig. 9. To guarantee a fixed time interval of 1-day, all the missing values in S-1 and S-2 features are interpolated, as shown in Fig. 10. The data is processed in batches of size $64 \times 215$ $\times$14, where 64 is the number of fields considered per patch, 215 is the number of days in the vegetation season in Estonia, 14 is the number of selected features.

The network’s output is a vector of size 215, representing the probability of a mowing event on each day in the vegetation season. The network consists of three one dimension convolution layers. The first and second convolution layers are followed by the Softmax activation function and batch normalization layer, while the third convolution is followed by Sigmoid activation function. The NN hyperparameters required to achieve the model learning process can significantly affect model performance. These hyperparameters include the following^56^:

- *Number of epochs* represents how many times you want your algorithm to train on your whole dataset.
- *Loss function* represents the prediction error of Neural Network.
- *Optimizer* represents algorithm or method used to change the attributes of the neural network such as weights and learning rate to reduce the loss.
- *Activation function* is the function through which we pass our weighted suown to have a significant output, namely as a vector of probability or a 0–1 output.
- *Learning rate* refers to the step of backpropagation, when parameters are updated according to an optimization function.

**Figure 11:**
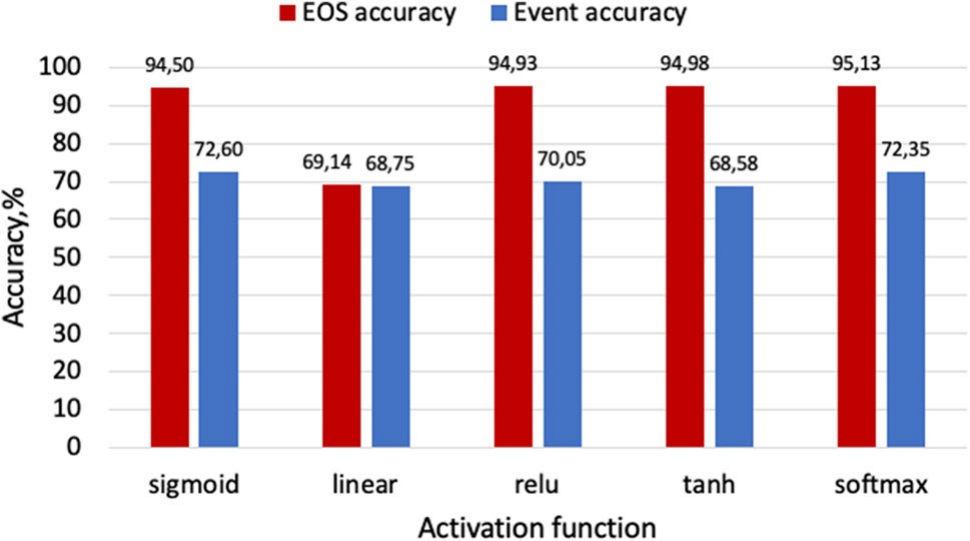
Performance of different activation functions.

A good model uses the optimal combination of these hyperparameters and achieves good generalization capability. The training was performed with the conjugate gradient descent method and the binary cross-entropy loss function. The neural network was trained during 300 epochs; an early stopping was used^59^. The optimizer used in our model is Nadam optimizer^60^ with the following parameters: $beta_1=0.9$, $beta_2=0.999$, $epsilon=None$, $schedule_{decay}=0.004$, and *learning* $rate=0.001$. Different activation functions such as ReLU, Sigmoid, Linear, and Tanh have been experimentally evaluated on the testing dataset as shown in Fig. 11. The results show that the Softmax activation function achieves the highest combined performance (event accuracy of 72.6% and EOS of 94.5%), as shown in Fig. 11.

Using 1*D* convolution layer acts as a filter that slides on the time dimension allowing the model to predict future mowing events from past events. However, this approach is not suitable for real-time detection of mowing events, but we use it to predict mowing events within a fixed time frame (window). Such a time frame should be greater than half the $1-D$ convolution window length.

#### Model evaluation

To evaluate our model, we used two metrics, EOS accuracy and Event-based accuracy. EOS is the accuracy of detecting a mowing event at least once during the season. If the probability of detecting a mowing event at least once during the season is more than 50%, then the field is considered mown, otherwise not mown. Event-based accuracy is used to evaluate how well our model correctly predicts mowing events. The formula for quantifying the binary accuracy is defined as follows:

9 $$\begin{aligned} acc = \frac{TP + TN}{TP + TN + FP + FN} \end{aligned}$$

where TP is the number of times that the model correctly predicted mowing events, given that the start day of the predicted mowing event is not more than 3 days earlier and not more than 6 days later than the actual start day of the mowing event. Within these 9 days, several mowing events may be predicted. To handle this case, only the first predicted mowing event is considered TP, and every next one is considered an FP. TN is the number of times that the model correctly predicted the absence of mowing events. FP is the number of times that the model incorrectly predicted mowing events. It also includes the number of times that the model correctly predicted mowing events, but the start of the event does not fit into a 9-days time frame with the actual start of some mowing event. FN is the number of times where the model missed actual mowing events.

#### Reject region

**Figure 12:**
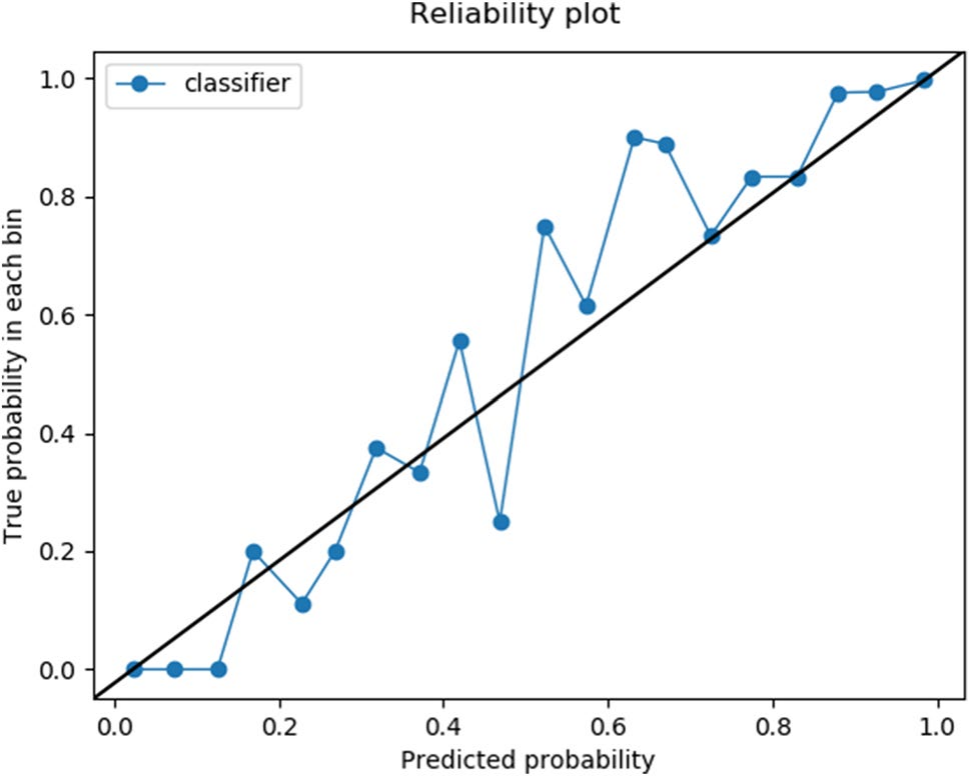
Calibration plot for proposed model.

Sometimes the model is not confident enough to give a reliable decision about the state of the field. We cannot expect reliable and confident predictions from inaccurate, incomplete or uncertain data. So, it is better in the cases of uncertainty about the prediction to allow the model to abstain from prediction. In this way, the obtained predictions are more accurate, while human experts could check rejected fields. Given the true positive rate and the true negative rate on the validation set, the reject region technique outputs a probability interval ($t_{low}$, $t_{upper}$) in which the model abstain prediction, where $t_{low}$ and $t_{upper}$ are the minimum and maximum probabilities that the model is uncertain about its prediction. Out of this interval, the model is confident about its prediction and predicts afield as mowed if the probability is higher than $t_{upper}$ and not mown if the probability is less than $t_{low}$. We select $t_{upper}$, such that the desired true positive rate is reached. To find $t_{upper}$, we sort all positives descending by their predicted probabilities and select the top percentage equal to the true positive rate. We choose $t_{low}$ such that the desired true negative rate on validation data is reached. To find $t_{low}$, we sort all negatives ascending by their predicted probabilities and select the top percentage equal to the true negative rate.

Figure 12 shows the calibration plot for our proposed model. Notably, the predicted probabilities are close to the diagonal, which implies that the model is well-calibrated.

## Results

### Neural network model optimization

**Figure 13:**
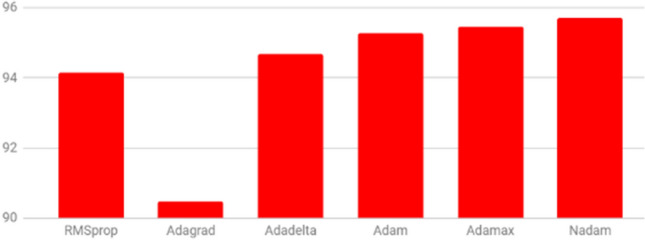
EOS accuracy for different optimizers.

**Figure 14:**
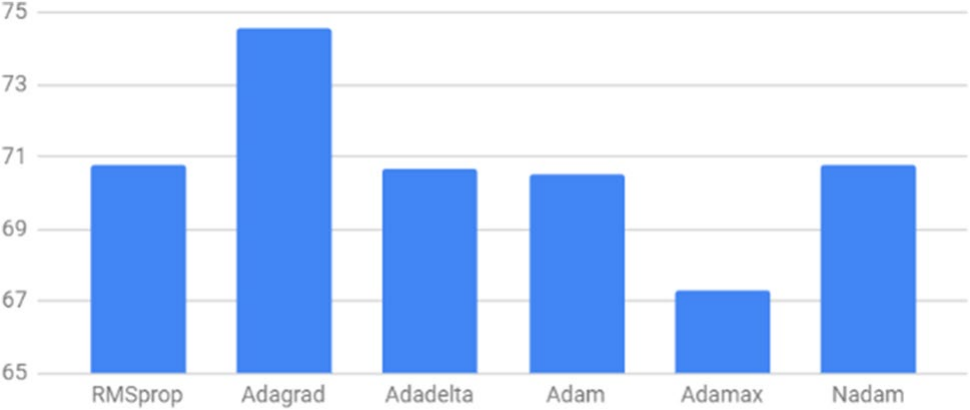
Event accuracy for different optimizers.

**Figure 15:**
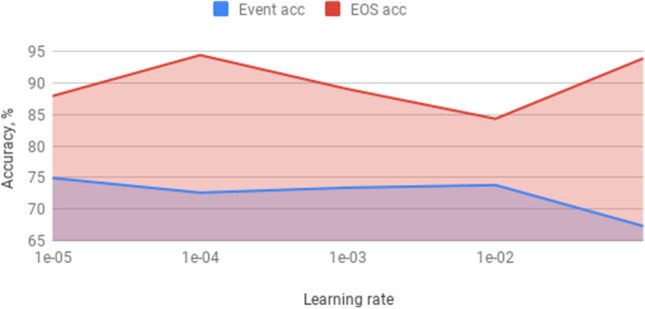
Performance using different values for learning rate parameter for Nadam optimizer.

We evaluated the performance of the proposed network using 6 different optimizers, including Adagrad, RMSprop, Adadelta, Adam, Adamax and Nadam using their default parameters. Figures 13 and 14 show the EOS accuracy and event accuracy, respectively, for the previous mentioned optimizers. Figure 13 shows that the EOS accuracy using Nadam optimizers slightly outperforms other optimizers. The Adagrad optimizers achieve the highest event accuracy, while Adamax achieves the lowest event accuracy, as shown in Fig. 14. For optimizing both of the metrics, Nadam optimizer is considered the best as it comes in the first place for the EOS accuracy and second place for the event accuracy, as shown in Figs. 13 and 14 . We used the default parameters for the Nadam optimizer except for the tuned learning rate. Figure 15 shows the performance of the network using Nadam optimizer using different values for the learning rate. The results show that choosing the learning rate value of $1e^{-4}$ achieves the best performance for both EOS accuracy and event accuracy. The Nadam optimizer parameters beta1 and beta2 have been experimentally evaluated, and results show that the default parameters for $beta1=0.9$ and $beta2=0.999$ achieve the best performance.

### Baseline

We compared the performance of the proposed neural network model to a multi-layer perceptron network used for automatic grassland cutting status detection^61^. The multi-layer perceptron network consists of an input layer connected to a layer of 31 hidden nodes, which are in turn connected to the output layer. To provide a fair comparison, we used the same training (64%), testing (16%), and validation (20%) splits on our proposed approach and the baseline network. In addition, the reject region technique is not considered in the comparison. Quantitatively, we present the event accuracy, end of season accuracy and AUC-ROC for our network compared to the network of Taravat et al.^61^, as shown in Table 4. The reported results in Table 4 are based on the same ground, as Taravat et al.^61^. The performance of the proposed approach outperforms the baseline and achieves event accuracy of 73%, EOS accuracy of 94.8% and AUC-ROC of 97%. The best overall performance (combined metrics) is marked in bold.

**Table 4:** Performance comparison between our network and other approaches.

	Event accuracy (%)	End of season accuracy (%)	AUC-ROC
Taravat et al.^61^	24	94.7	95
Proposed approach	**73.3**	**94.8**	**97**

### Reject region performance

The performance of the reject region technique is tested with the three different configurations of desired precision and recall values. Table 5 summarizes the performance of the different precision and recall configurations. The results show that setting the precision and recall to 90% improves the AUC compared to not setting any configuration. Using the configuration of precesion = 97% and recall = 75% achieves the highest overall performance (accuracy = 92.4% and AUC = 97%) with 12.8% rejected instances in which the model is uncertain. The best overall performance (combined metrics) for a particular configuration is marked in bold.

**Table 5:** Reject region summary performance of three different configurations.

Recall (%)	Precision (%)	Accuracy (%)	AUC	($t_{low}$,$t_{upper}$)	% of Rejected instances
–	–	92.4	0.94	–	0
90	90	86.5	0.99	(0.04, 0.78)	24.8
97	75	80.7	0.96	(0.13, 0.86)	30.0
75	97	**92.4**	**0.97**	(0.04, 0.52)	12.8

## Discussion

  Weather conditions significantly impact the quality of features extracted from Sentinel images. A decreasing trend in coherence values is observed before a mowing event. Some factors impeded the increase of coherence after mowing events, such as rapid grass growth and precipitation before image acquisitions. Rainy weather remarkably decreases the coherence values. Generally, the typical processing pipelines of different land monitoring applications depends, among many other features, on the NDVI feature for a single date or a whole . We noticed that single-date cloudy observations resulted in a sudden drop in the NDVI that can easily lead to overestimating the mowing events. It is also notable that the NDVI feature and many spectral features are unavailable under cloudy weather.

Sentinel data may significantly impact several vital earth monitoring applications, such as ice monitoring, climate change monitoring, agriculture and forestry planning, map updating, flood monitoring. The proposed machine learning pipeline we developed can easily be transferred to different earth monitoring applications. This study is a proof of concept for using Sentinel-1 images and Sentinel-2 images for grassland management. We analyzed the performance of the proposed machine learning pipeline. Notably, most of the incorrectly predicted events have the following characteristics: (1) the number of Sentinel-2 images for the fields is less than 10 while the number of Sentinel-1 images is roughly the same for all fields, (2) the size of the field is significantly less than average, e.g., lower than 1ha, which means that only several pixels were aggregated to receive feature values, (3) fields that are located in extreme areas, e.g., valleys; these fields could be highly wet or even flooded during the vegetation seasons, which causes feature outliers and often incorrect predictions. The main limitation of our proposed approach is the sensor spatial resolution that is too coarse for imaging narrow and long fields for rural areas in different mountainous areas^62,63^. Such limitation makes the proposed approach inapplicable for cases when a high level of detail is required for deciding small spatial scales. A feasible alternative in such cases is unmanned aerial vehicles. The continued acquisition of Sentinel-1 images in the future will facilitate the construction of longer time series that can lead to better detection of agriculture events.

## Conclusion

Recent advances in SAR remote sensing create new natural environment applications such as forest floods and agricultural grasslands. The goal of this study was to develop a model for the automatic identification of mowing events. A cost-effective solution is presented, capable of processing supertemporal acquisitions covering Estonian grasslands throughout the whole farming season. Although optical data are most suited for identifying grasslands from other land cover types, they are subject to changing weather conditions, which hinder the ability to collect adequate information over the complete phenological cycle. SAR images are incorporated to handle this limitation as they are independent of weather conditions. In this work, we investigate the performance of *S*-1 and *S*-2 images, covering 2000 fields in Estonia in 2018, to detect grass mowing events. Our proposed approach first preprocess both S-1 and S-2 images and then incorporates the a set of derived features into a deep learning model to detect mowing events. Results show that the proposed model outperforms Taravat et al.^61^ approach and achieves event accuracy of 73.3% and end of season accuracy of 94.8%. In addition, the proposed model provides a reject region option that allows the model to abstain from the prediction in case of low confidence, which increases trust in the model. We experimentally evaluated different configurations for the reject region technique, and the results show that the best performance achieved is accuracy of 92.4% and area under the ROC curve 99%.
